# First Complete Mitochondrial Genomes of the Invasive Mussel *Perna viridis* from Brazil and the Southwestern Atlantic

**DOI:** 10.3390/biology15141199

**Published:** 2026-07-20

**Authors:** André Oliveira Souza Lima, Rafael Schroeder, Gabriela S. Delabary, Gilberto C. Manzoni, Mayara C. Beltrão

**Affiliations:** 1 Laboratório de Genética Molecular, Escola Politécnica, Universidade do Vale do Itajaí (UNIVALI), Rua Uruguai 458, Itajaí 88302-901, Brazil; gabrielad@univali.br; 2 Laboratório de Estudos Marinhos Aplicados, Escola Politécnica, Universidade do Vale do Itajaí (UNIVALI), Rua Uruguai 458, Itajaí 88302-901, Brazil; 3 Centro Interdisciplinar de Investigação Marinha e Ambiental (CIIMAR), Terminal de Cruzeiros do Porto de Leixões, Avenida General Norton de Matos S/N, 4550-208 Matosinhos, Portugal; 4 Laboratório de Maricultura, Escola Politécnica, Universidade do Vale do Itajaí (UNIVALI), Rua Uruguai 458, Itajaí 88302-901, Brazil; manzoni@univali.br; 5 Laboratório de Ecologia de Comunidades Aquáticas, Escola Politécnica, Universidade do Vale do Itajaí (UNIVALI), Rua Uruguai 458, Itajaí 88302-901, Brazil; beltrao@univali.br

**Keywords:** Mytilidae, Oxford Nanopore sequencing, comparative mitogenomics, gene order, mitochondrial reference sequences, marine biosecurity, aquaculture monitoring

## Abstract

The Asian green mussel *Perna viridis* is expanding across coastal systems of the Americas, creating demand for curated molecular references to support biosecurity, aquaculture monitoring, and comparative invasion studies. Here, we report the first complete mitochondrial genomes of invasive *P. viridis* from Brazil and the southwestern Atlantic, generated from two specimens collected in Santa Catarina, currently the southernmost documented sector of the Brazilian range. Long-range PCR, Oxford Nanopore sequencing, read-supported assembly, manual annotation, comparative mitogenomics, nucleotide-diversity analysis, protein-level divergence, and phylogenomic inference were used to generate and evaluate complete mitochondrial references. The two circular assemblies were 16,015 bp and 16,011 bp and contained 38 annotated features, including 13 protein-coding genes, 23 tRNAs, and two rRNAs, all on the H strand, with an identical A+T content of 67.5%. Pairwise comparisons among the four complete *P. viridis* mitogenomes analyzed showed high overall similarity but retained 20–125 SNPs/mismatches and one to 12 indel events. Gene order was conserved within *P. viridis* but differed from *P. canaliculus* and *P. perna*, and phylogenomics placed the Brazilian assemblies within the *P. viridis* clade. These curated mitogenomes provide initial complete mitochondrial references for future marker testing and comparative studies of *P. viridis* in the southwestern Atlantic, but the two-specimen dataset should not be used to infer population origin, invasion routes, or connectivity.

## Simple Summary

Researchers sequenced the first complete mitochondrial genomes of invasive Asian green mussels (*Perna viridis*) from Brazil. The two genomes are about 16,000 base pairs long, contain 38 genes, and have 67.5% A+T content. These genetic references provide complete mitochondrial sequences for future comparative studies of the mussel in the southwestern Atlantic.

## 1. Introduction

Biological invasions are a major driver of global environmental change, reshaping biodiversity patterns, community structure, ecosystem functioning, and biogeographic boundaries across aquatic and terrestrial systems [1,2]. In coastal environments, non-indigenous species are strongly facilitated by human-mediated connectivity, including maritime transport, port infrastructure, aquaculture, ballast water, hull fouling, artificial substrates, and recurrent vessel movement among regions [1,3]. Once established, aquatic invasive alien species can modify native communities, disrupt trophic and functional interactions, affect ecosystem services, and impose substantial costs on fisheries, aquaculture, infrastructure, conservation, and coastal management [4]. Because coastal invasions are often detected only after local establishment, accurate taxonomic identification and molecular surveillance have become increasingly important for marine biosecurity and monitoring [3,5,6].

Mytilid mussels are among the most successful marine invaders because their sessile, filter-feeding habit is coupled with efficient substrate colonization, strong byssal attachment, high local biomass, and the capacity to form dense aggregations on natural and artificial surfaces [7,8]. Within this group, the Asian green mussel *Perna viridis* (Linnaeus, 1758), native to tropical and subtropical Asian coastal waters, has expanded beyond its native range and is now established in multiple coastal regions of the Americas [8,9]. Its invasive performance has been associated with rapid biomass accumulation, tolerance to environmental variation, and frequent occupation of ports, marinas, aquaculture structures, piers, and other submerged anthropogenic substrates [8,9,10,11]. These attributes increase the potential for competition with native and cultivated bivalves, biofouling of coastal infrastructure, contaminant accumulation, and broader ecological and economic impacts in invaded systems [9,12].

In Brazil, *P. viridis* was first reported in Guanabara Bay, Rio de Janeiro, in 2018, representing the first documented occurrence of the species in the southwestern Atlantic [13]. Subsequent records from Rio de Janeiro, including Arraial do Cabo and additional sites in Guanabara Bay, together with more recent confirmations in São Paulo, Paraná, and Santa Catarina, indicate that the species is expanding along the Brazilian coast rather than remaining restricted to an isolated introduction event [9,14,15,16,17,18,19,20]. This expansion is particularly relevant in southern Brazil, where Santa Catarina represents the currently documented southern invasion front and includes one of the country’s most important mollusk-farming regions. The confirmation of *P. viridis* in the Itapocoroy Bay farming area is therefore especially significant, because it places the invader in a sector where biofouling pressure, aquaculture activities, and potential interactions with the cultivated mussel *Perna perna* may converge [19]. Together, these records reinforce the need for integrated taxonomic, ecological, and molecular monitoring of *P. viridis* along the Brazilian coast.

Molecular confirmation of Brazilian records has relied predominantly on morphology plus partial mitochondrial cytochrome c oxidase subunit I (*COX1*) sequences [9,15,19,20]. *COX1* barcoding remains indispensable for species-level identification, especially when juvenile or fouling specimens are difficult to distinguish morphologically or when closely related mytilids co-occur [19,20,21]. However, a single barcode fragment captures only a limited portion of mitochondrial variation, and marker choice can influence downstream biogeographic interpretation [22]. By itself, *COX1* does not resolve genome architecture, gene order, annotation boundaries, strand distribution, or the location of more variable regions across the mitochondrial genome [23].

Complete mitogenomes therefore add mitochondrial characters for invasive-range studies of *P. viridis*. Beyond taxonomic confirmation, they enable comparisons of gene content and order, coding-region boundaries, codon usage, intergenic organization, and spatially heterogeneous variation across the mitochondrial genome, while also improving datasets for phylogenomic placement, marker selection, and future comparative analyses [5,23,24]. Such information is particularly relevant in Mytilidae, where mitochondrial organization can vary substantially among lineages and careful annotation is required for reliable comparative inference [23,24,25].

Despite this value, public mitogenomic resources for *Perna* remain sparse. A curated NCBI survey recovered only 15 complete records for the genus and only two non-redundant mitogenomes assigned to *P. viridis*, indicating that the currently available complete mitogenome dataset for the species is limited. This limitation is important because congeneric *Perna* mitogenomes can differ in organization and annotation, making new complete and carefully curated references from invaded regions necessary for consistent comparison [24,26,27].

In this study, we report the first complete mitochondrial genomes of *P. viridis* from Brazil, generated from two invasive specimens collected in Santa Catarina. By characterizing their mitogenome architecture and comparing them with the complete *Perna* mitogenomes currently available in public databases, we aim to provide two curated complete mitochondrial references for future comparisons of *P. viridis* in the southwestern Atlantic. Given the two-specimen Brazilian sampling and the still-limited mitogenomic sampling available for the species, our objective is not to infer geographic origin, invasion routes, or population structure, but to provide a reference sequence for subsequent work on marker development, broader phylogeographic comparison, and population-level investigation.

## 2. Materials and Methods

### 2.1. Sample Collection, DNA Extraction, and Long-Range PCR

Muscle tissue from two *P. viridis* specimens collected in Florianópolis, Santa Catarina, Brazil, in 2025 was used for complete mitochondrial genome sequencing. The specimens were registered as LGM-781 and LGM-885 and originated from two coastal locations in Florianópolis: 27°30′31.0″ S, 48°31′10.1″ W and 27°44′41.95″ S, 48°33′44.31″ W, respectively [20]. The *COX1*-based molecular identification and genus-level *Perna* phylogenetic placement of these same specimens were reported previously by Beltrão et al. [20]; the present study focuses on complete mitochondrial genome sequencing and comparative mitogenomics. Access to the Brazilian genetic heritage associated with these specimens was registered in SisGen under registration number AE5545E. Approximately 25 mg of muscle tissue from each specimen was used for DNA extraction. Total DNA was extracted using the DNeasy Blood & Tissue Kit (Qiagen, Hilden, Germany) according to the manufacturer’s protocol and quantified by spectrophotometry at 260/280 nm using a Tecan Infinite M200 microplate reader (Tecan Group Ltd., Männedorf, Switzerland). Only DNA with A260/280 values between 1.8 and 2.0 and a minimum concentration of 20 ng/µL was used for downstream steps.

To obtain a complete mitogenome, the available *P. viridis* mitochondrial reference sequence (NC_018362.1 [26]) was used to guide the design of two overlapping long-range PCR targets. Primer pairs were developed by the authors using an in-house Python (v3.14.6) script based on Primer3-assisted primer design [28], followed by the manual inspection of candidate pairs. The selected primers targeted two mitochondrial fragments centered on the *ND4* and *ND5* regions, respectively, and were chosen to provide broad mitogenome coverage while maintaining suitable oligonucleotide length, melting temperature, GC content, and low predicted primer-dimer formation. UGENE (v49.1 [29]) was used to visualize primer placement along with the reference sequence. Primer sequences are provided in Table 1. Detailed primer properties, including oligonucleotide length, GC content, melting temperature, predicted dimer/hairpin metrics, reference coordinates, and theoretical amplicon lengths, are provided in Supplementary Table S1. Sample-identifying 5-nt barcodes designed in-house were appended to the 5′ ends of primers to enable multiplex sequencing and post-run demultiplexing.

Long-range PCRs were performed in 25 µL reactions containing 4 µL of DNA template (50 ng total DNA per reaction), 1× buffer (Thermo Fisher Scientific, Waltham, MA, USA), 0.2 mM dNTPs (Promega, Madison, WI, USA), 0.5 µM of each primer, and 0.25 U of Platinum™ SuperFi II DNA Polymerase (Thermo Fisher Scientific, Waltham, MA, USA). Thermal cycling was performed on a Kasvi thermocycler (model K33-10TG, Kasvi, Pinhais, PR, Brazil) using the following program: initial denaturation at 98 °C for 50 s; 35 cycles of 98 °C for 15 s, 57 °C for 35 s, and 72 °C for 5 min 30 s; followed by a final extension at 72 °C for 12 min. Amplicons were checked by electrophoresis in 0.8% agarose gel prepared in 1× TAE, using a λ DNA/HindIII molecular marker (Thermo Fisher Scientific, Waltham, MA, USA), and visualized under UV; gels were documented using EOS Utility (Canon Inc., Tokyo, Japan).

### 2.2. Oxford Nanopore Sequencing, Read Processing, and Mitogenome Assembly

Long-range PCR amplicons were purified using the Wizard^®^ SV Gel and PCR Clean-Up System (Promega, Madison, WI, USA) and prepared for Oxford Nanopore sequencing using the Ligation Sequencing Kit SQK-LSK114 (Oxford Nanopore Technologies, Oxford, UK). End-repair of DNA fragments was performed using enzymes from the Companion Module for Oxford Nanopore Technologies^®^ Ligation Sequencing (New England Biolabs, Ipswich, MA, USA), according to the manufacturer’s protocols. Libraries were loaded onto Flongle flow cells (FLO-FLG114, Oxford Nanopore Technologies, Oxford, UK) and sequenced on a MinION device (Mk1B, Oxford Nanopore Technologies, UK) running MinKNOW (v25.03.9, Oxford Nanopore Technologies, Oxford, UK). Basecalling was performed with Dorado v2.1.0 (Oxford Nanopore Technologies, Oxford, UK) in super-accurate mode, applying a minimum read quality threshold of Q ≥ 8 and generating FASTQ files.

FASTQ reads were processed using a custom Python script to demultiplex barcoded libraries based on 5-nt tags appended to the 5′ ends of the amplification primers. Because the sequencing run included multiple multiplexed libraries, assembly-level quality control was based on quality-filtered and demultiplexed reads retained for each amplicon, with read-level support metrics summarized in Supplementary Table S2. Reads were clustered using CD-HIT v4.8.1 [30], aligned with MAFFT v7.520 [31], and consensus sequences were generated using EMBOSS v6.6.0.0 [32].

For each *P. viridis* specimen, the highest-supported consensus sequence from each mitochondrial fragment was selected for final assembly, resulting in two long-amplicon consensuses per specimen. These consensuses were processed using an in-house Python workflow that identified terminal overlaps using a minimum overlap of 300 bp, ≥95% identity, and ≥50% comparable bases. Fragments were merged by majority rule, with ambiguous positions assigned as N when base support was insufficient. Conflicts in overlapping regions were resolved using the original long reads through a seed-based search with reverse-complement matching, a 15 bp flank, a 13 bp seed size, a maximum mismatch threshold of 18%, and up to 500 supporting reads per position. The merged assemblies were then compared with available *P. viridis* mitochondrial references from NCBI using minimum thresholds of 95% identity and 95% coverage. Redundant terminal overlap generated during linear assembly of the circular mitogenome was trimmed using the same overlap criteria, and the curated sequences were retained as complete mitochondrial genome assemblies for downstream analyses. Key workflow steps, external tools, input/output files, thresholds, and parameters used by the custom read-processing and assembly scripts are summarized in Supplementary Table S3.

### 2.3. Mitogenome Annotation, Diversity Metrics, and Phylogenetic Analysis

Complete mitogenome consensus sequences were validated by similar searches using BLASTn v2.17.0+ (NCBI, Bethesda, MD, USA) against the NCBI Core nucleotide database to confirm taxonomic identity [33]. Pairwise comparisons among complete *P. viridis* mitogenomes were performed with an in-house Python script, calculating identity from comparable A/C/G/T sites and recording SNPs/mismatches and consecutive-gap indel events. Mitogenomes were annotated through reference-assisted nucleotide-level comparison with the *P. viridis* RefSeq mitogenome NC_018362.1, followed by manual curation of gene boundaries and coding regions; coding sequences were checked using the invertebrate mitochondrial genetic code (Table S1). The annotation was not transferred automatically from the reference; gene boundaries, reading frames, start and stop codons, circular continuity, and intergenic/overlap intervals were inspected manually. Gene features were extracted to generate annotation tables including gene name, coordinates, length, and strand, covering protein-coding genes, tRNAs, rRNAs, and non-coding regions when present. Circular genome representations were generated with the MitoDraw module of MitoFish [34]. Gene-order organization was represented schematically from GenBank (GBK) annotations by structuring CDS, tRNA, and rRNA features as comparative blocks [24]. Software versions, workflow steps, and key parameters used for reading processing, assembly, annotation, comparative analyses, and phylogenetic reconstruction are summarized in Supplementary Table S3.

Relative synonymous codon usage (RSCU) was computed from the extracted protein-coding genes in PhyloSuite v1.2.3 [35,36,37]. For each mitochondrial protein-coding gene, amino acid sequences were extracted from the complete *Perna* mitogenomes in the comparative dataset (Supplementary Table S4), aligned separately, and used to calculate mean pairwise amino acid divergence across comparable positions [38,39,40,41,42,43]. Genome-wide nucleotide diversity (π) was estimated from an *ATP6*-anchored alignment of the complete *P. viridis* mitogenomes analyzed in this study. Sequences were aligned with Clustal Omega v1.2.4 (DNA mode; --auto) [44], and sliding-window π was computed with a custom Python script written in Python v3.14.6 (window = 500 bp; step = 25 bp). For each window, π was calculated from pairwise differences per site after excluding alignment columns with >50% gaps and retaining only positions with valid DNA calls (A/C/G/T) in at least 50% of sequences. Within each retained window, π was computed as the mean proportion of nucleotide differences across all pairwise sequence comparisons and retained sites.

For phylogenetic placement, the 13 mitochondrial protein-coding genes were extracted from each GBK file and aligned separately for all mitogenomes listed in Supplementary Table S4. Non-coding/intergenic intervals were not included in this phylogenetic dataset because reliable positional homology across rearranged congeneric mitogenomes could not be established confidently. The gene alignments were concatenated in the order *COX1*, *COX2*, *COX3*, *ATP6*, *ATP8*, *CYTB*, *ND1*, *ND2*, *ND3*, *ND4*, *ND4L*, *ND5*, and *ND6*, yielding a final nucleotide alignment of 15 sequences and 12,051 positions. The dataset included *Mytilus galloprovincialis* OR574991.1 as the outgroup. Maximum-likelihood phylogenetic inference was performed with RAxML-NG v2.0.2 [45] using a gene-partitioned dataset with 13 partitions, GTR + FC + G4m models estimated for each partition, three starting trees, and 1000 Felsenstein bootstrap replicates [46]. The best-scoring ML tree was visualized in iTOL v6 [47] and rooted using the same outgroup.

### 2.4. Use of Artificial Intelligence Tools

Artificial intelligence tools (ChatGPT, GPT-5.5 Thinking, OpenAI, San Francisco, CA, USA) were used during manuscript preparation to assist with translation, English-language refinement, editorial organization, and text revision. No artificial intelligence tool was used to generate, alter, or manipulate the original research data, results, analyses, or figures. All content was reviewed and verified by the authors, who take full responsibility for the final manuscript.

## 3. Results

### 3.1. Sampling Sites Within the American Invasion Context

**Figure 1:**
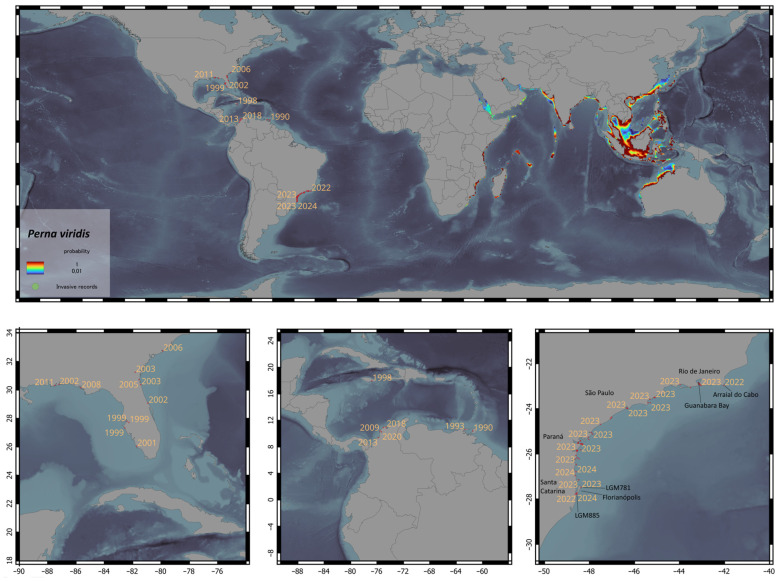
Mapped non-native *Perna viridis* occurrence records in the Americas and sampling sites of the Brazilian mitogenomes generated in this study. Red points indicate curated occurrence records retained after taxonomic and bibliographic screening, and labels indicate the year assigned to each record. The upper panel shows the native range and global distributional context of *P. viridis* together with the mapped American records. In contrast, the lower panels provide regional views of North America, Central America/Caribbean, and South America. Brazilian records are concentrated along the southeastern and southern coasts, including the Santa Catarina sector from which LGM-781 and LGM-885 were obtained. Coordinates, year-assignment criteria, sources, and confidence notes for all mapped records are provided in Supplementary Table S5. Brazilian specimens are geographically represented but should not be interpreted as evidence of source population, invasion route, or number of introduction events.

The curated occurrence matrix used to construct Figure 1 included 84 mapped non-native records of *P. viridis* in the Americas, retained after taxonomic and bibliographic screening of sources available up to May 2026, with occurrence years ranging from 1990 to 2024. Sources and mapped locations are summarized in Supplementary Table S5 [12,13,14,16,17,18,19,20,43,48,49,50,51,52,53,54,55]. The records indicate a broad but discontinuous western Atlantic distribution, with early occurrences in the Caribbean followed by detections along the southeastern United States and Colombian Caribbean, consistent with a regional invasion history shaped by human-mediated coastal connectivity [12,43,50,51,52,53,54,55,56].

Brazilian records constituted the largest subset of the curated matrix, with 64 mapped occurrences along the southeastern and southern coasts. The first Brazilian record occurred in Guanabara Bay, Rio de Janeiro, in 2018, which was followed by detections in other areas of Rio de Janeiro and, more recently, in São Paulo, Paraná, and Santa Catarina. Most Brazilian records were assigned between 2022 and 2024, highlighting the recent intensification of documented occurrences along the coastal sector extending from Rio de Janeiro to Santa Catarina [9,14,16,17,18,19,20].

The two specimens sequenced here, LGM-781 and LGM-885, were collected in Florianópolis, Santa Catarina, Brazil, at 27°30′31.0″ S, 48°31′10.1″ W and 27°44′41.95″ S, 48°33′44.31″ W, respectively. These sites are within the southern portion of the documented Brazilian invasion range and are of utmost relevance for mollusk aquaculture and coastal biosecurity. Accordingly, the mitogenomes generated here provide complete mitochondrial sequences from the southern Brazilian invasion front. At the same time, the geographic distribution summarized in Figure 1 should not be interpreted as evidence of source population, invasion route, or number of introduction events.

### 3.2. Sequencing Output, Mitogenome Assembly, and Comparative Validation

The two-primer-pair long-range PCR strategy generated mitochondrial consensus sequences for both *P. viridis* specimens and target regions. Selected long-amplicon consensuses ranged from 8459 to 8659 bp and were supported by 956–3918 demultiplexed Nanopore reads per fragment (Table 2). The similar length of the amplicons resulted in an approximate mean fragment-level read support of ~2636 reads for LGM-781 and ~1365 reads for LGM-885. In both specimens, overlapping consensuses enabled complete mitogenome reconstruction; after overlap-based merging, read-supported conflict resolution, and trimming of terminal redundancy generated by circular linearization, final assemblies measured 16,015 bp for LGM-781 and 16,011 bp for LGM-885. The complete mitochondrial genome assemblies were deposited in GenBank under accession numbers PZ507420 for LGM-781 and PZ507421 for LGM-885.

Both primer pairs generated the expected overlapping mitochondrial fragments in both specimens, supporting complete mitogenome assembly (Table 2; Supplementary Table S1). BLASTn validation supported the assignment of both final assemblies to *P. viridis*, with the highest-scoring matches corresponding to publicly available *P. viridis* mitochondrial sequences. Detailed BLASTn outputs for LGM-781 and LGM-885 are provided in Supplementary Tables S6 and S7, and the direct pairwise BLAST comparison between the two Brazilian mitogenomes is provided in Supplementary Table S8. Full-mitogenome pairwise comparisons among the complete *P. viridis* records analyzed showed 99.219–99.875% identity across comparable A/C/G/T sites (Table 3). Together with the two Brazilian mitogenomes generated here, the two public complete *P. viridis* mitogenomes included in the comparative analyses formed a four-sequence complete-mitogenome dataset for the species. The public records were NC_018362.1, from China and associated with Li et al. [26], and MW727515.1, from Australia and listed in GenBank without an associated publication record. These metadata are provided in Supplementary Table S4.

Despite this high similarity, the comparisons retained 20–125 SNPs/mismatches and 1–12 indel events and 1–17 indel bases. LGM-781 and LGM-885 differed by 125 SNPs/mismatches and nine indel events, and each Brazilian mitogenome showed a different closest match among complete public *P. viridis* accessions. These pairwise patterns were used to validate the assemblies in a comparative context. Given the limited number of complete *P. viridis* mitogenomes currently available, the observed relative similarities should not be interpreted as evidence of source population, invasion route, or number of introduction events.

### 3.3. Mitogenome Architecture and Curated Annotation

**Figure 2:**
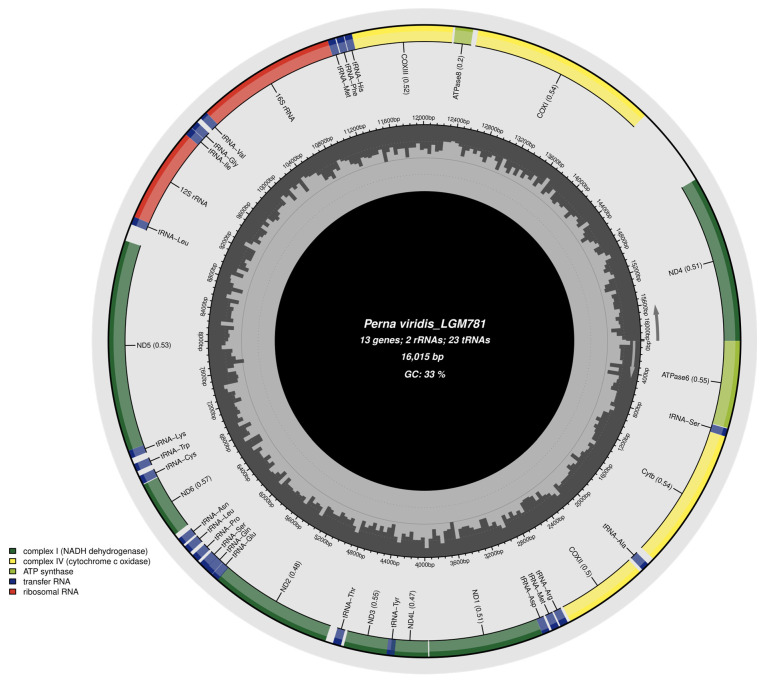
Circular map of the representative *Perna viridis* mitochondrial genome LGM-781 (16,015 bp; G+C = 32.5%). The outer ring shows annotated features and orientation along the circular molecule; colors indicate complex I/NADH dehydrogenase genes (dark green), complex IV/cytochrome c oxidase genes (yellow), ATP synthase genes (light green), tRNAs (blue), and rRNAs (red). The inner histogram depicts local nucleotide-composition variation. *ND4* is represented as an origin-spanning feature because the coding sequence crosses the artificial coordinate break of the circularized mitogenome and overlaps the *ATP6*-start interval.

The two Brazilian *P. viridis* mitogenomes had the same annotated gene complement and overall circular architecture. Each assembly contained 38 mitochondrial features, namely 13 protein-coding genes, 23 tRNAs, and two rRNAs, all encoded on the H strand under the selected orientation. The major non-coding/intergenic region was located between *COX1* and *ND4* and measured 661 bp in LGM-781 and 660 bp in LGM-885. The assemblies differed by four base pairs in total length but showed identical A+T content (67.5%) and closely matched compositional skew values (Table 4). Because of this architectural similarity, LGM-781 is presented as the representative circular map (Figure 2), whereas Table 4 summarizes the compositional and structural features of both assemblies.

Coordinate-level annotation confirmed the same feature set in both Brazilian specimens, with minor coordinate differences corresponding to the four-base length difference and local indel variation. Complete gene coordinates, feature lengths, initiation and termination codons for protein-coding genes, and strand assignments are provided in Supplementary Tables S9 and S10. In both mitogenomes, *ND4* was annotated as a single origin-spanning protein-coding gene. Under the *ATP6*-start representation, *ND4* crosses the artificial coordinate break and terminates within the first bases of the *ATP6* interval; reporting it with split circular coordinates preserves coding-sequence continuity, including its terminal codon, and avoids truncation at the linearized genome boundary [56].

### 3.4. Gene-Order Conservation Within Brazilian P. viridis and Rearrangement Among Perna

**Figure 3:**
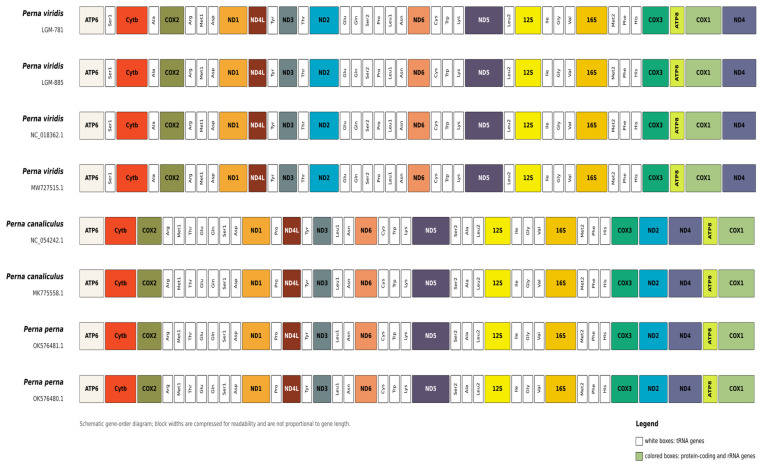
Schematic representation of mitochondrial gene order among selected mitogenomes of *P. viridis*, *Perna canaliculus*, and *Perna perna*. Labels on the left show taxon names, and public GenBank/NCBI accession codes and sample identifiers used in this study. Gene names are shown inside each block. White boxes represent tRNA genes, whereas colored boxes represent protein-coding and ribosomal RNA (12S and 16S) genes. All records are shown using an *ATP6*-start representation for direct comparison. Block widths are schematic and were adjusted to improve readability; therefore, they are not proportional to gene length or genomic distance.

Gene-order comparison showed complete conservation among the two Brazilian *P. viridis* mitogenomes and the two publicly available complete *P. viridis* records included in the analysis (Figure 3; Supplementary Table S4).

All four mitogenomes shared the same *ATP6*-start arrangement, supporting the structural consistency of the Brazilian assemblies within the currently available complete-mitogenome dataset for the species. By contrast, comparisons with *P. canaliculus* and *P. perna* revealed marked interspecific differences in mitochondrial gene order within *Perna*, particularly in the relative positions of *ND2*, *ND4*, *ATP8*, *COX1*, and adjacent tRNA blocks. Thus, although the Brazilian assemblies did not reveal a novel gene-order variant within *P. viridis*, the genus-level comparison demonstrates that complete mitogenomes capture structural information that cannot be recovered from single-locus markers.

### 3.5. Codon Usage, Nucleotide Diversity, and Protein-Level Divergence

**Figure 4:**
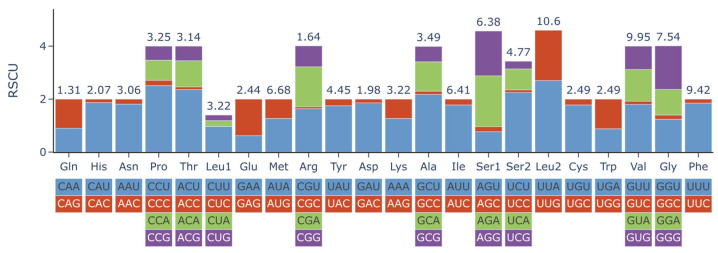
Relative synonymous codon usage (RSCU) profile of the representative Brazilian *Perna viridis* mitogenome LGM-781. The analysis was based on the 13 annotated mitochondrial protein-coding genes. Stacked bars summarize codon usage for each amino acid class, with codon labels shown below the corresponding bars.

The relative synonymous codon usage (RSCU) profile of the representative Brazilian mitogenome LGM-781 summarized codon-use composition across the 13 mitochondrial protein-coding genes (Figure 4). Because LGM-781 and LGM-885 had the same annotated gene complement and highly similar mitogenome architecture, a single representative RSCU profile was used to avoid redundant presentation. This profile provides a gene-set-level view of codon-use heterogeneity across mitochondrial amino acid classes and complements the nucleotide-composition metrics reported in Table 4. The profile also shows that synonymous codon use is not uniform among codon families, consistent with the A+T-rich composition of the mitochondrial genome, and provides a descriptive reference for future comparisons of codon-use patterns in *P. viridis*.

**Figure 5:**
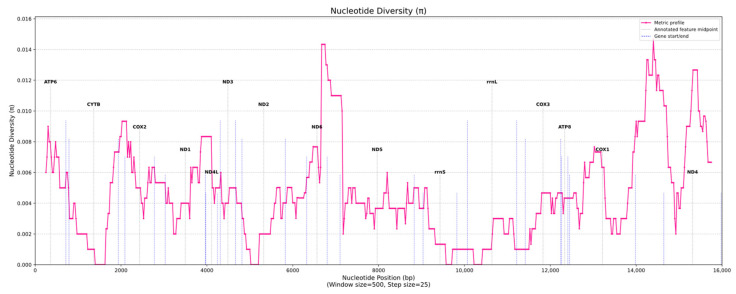
Sliding-window nucleotide diversity (pi) across complete *Perna viridis* mitochondrial genomes. The analysis included the two Brazilian assemblies generated in this study and the two publicly available complete *Perna viridis* mitogenomes listed in Supplementary Table S4. Diversity was calculated using a 500 bp window and a 25 bp step along the *ATP6*-start mitogenome alignment. Gene labels provide positional landmarks along the mitochondrial genome.

Sliding-window analysis of nucleotide diversity across the four complete *Perna viridis* mitogenomes revealed a heterogeneous distribution of variation along the mitochondrial genome rather than a uniform genome-wide signal (Figure 5). Diversity remained low across broad intervals but increased in discrete regions, including portions of the *ND6*/*ND5* region and the *COX1*–*ND4* sector. These variable windows identify mitogenomic regions that may guide future marker testing, without implying population structure or invasion routes from the current dataset.

**Figure 6:**
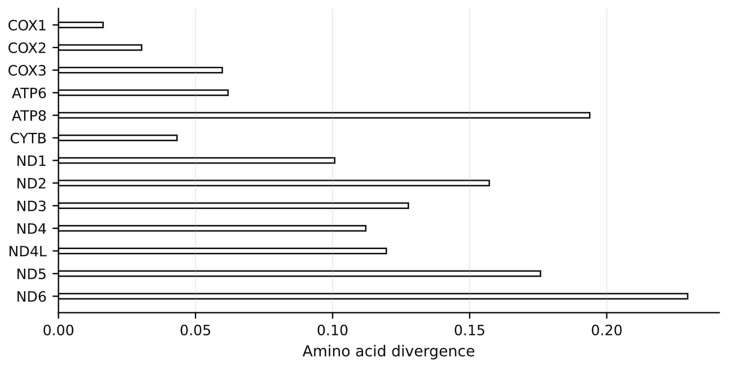
Mean amino acid divergence by mitochondrial protein-coding gene across *Perna* mitogenomes. The analysis included 14 *Perna* mitogenomes representing three species (*P. viridis*, *P. canaliculus*, and *P. perna*): the two Brazilian assemblies generated here and 12 public *Perna* mitogenomes listed in Supplementary Table S4. Amino acid sequences were extracted from the annotated mitochondrial protein-coding genes, aligned by gene, and compared across the *Perna* mitogenomes listed in Supplementary Table S4. Lower values indicate more conserved proteins, whereas higher values indicate greater protein-level divergence across comparable amino acid positions.

Protein-level comparisons across *Perna* mitogenomes showed a similarly uneven pattern of divergence among mitochondrial protein-coding genes (Figure 6). *COX1* and *COX2* were among the most conserved proteins, whereas *ATP8* and *ND6* showed the highest means of amino acid divergence, with additional elevated divergence in *ND5*, *ND2*, *ND3*, *ND4L*, and *ND4*. Together, the codon-usage, nucleotide-diversity, and protein-divergence analyses indicate that complete mitogenomes provide complementary information on conserved and variable mitochondrial features relevant to future marker testing and comparative studies.

### 3.6. Phylogenomic Placement of Brazilian P. viridis Mitogenomes

**Figure 7:**
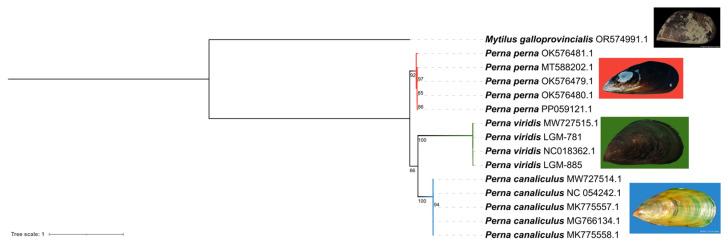
Phylogenomic placement of the Brazilian *Perna viridis* mitogenomes generated in this study. The Maximum Likelihood tree was inferred with RAxML-NG from a gene-partitioned concatenated nucleotide alignment of 13 mitochondrial protein-coding genes from complete mitogenomes of *Perna* and the outgroup *Mytilus galloprovincialis* OR574991.1 [38], listed in Supplementary Table S4. The *P. viridis* subset comprised the two Brazilian assemblies generated here, LGM-781 and LGM-885, and two complete public records, NC_018362.1 from China [26] and MW727515.1 from Australia [41]. The final alignment comprised 15 sequences and 12,051 positions. Node values indicate Felsenstein bootstrap support from 1000 replicates [46]. The Brazilian assemblies LGM-781 and LGM-885 cluster within *P. viridis* and are separated from *P. canaliculus* [27,39,40] and *P. perna* [24,41,42], supporting their taxonomic assignment as *P. viridis* and their inclusion in species-level mitogenome comparisons. Figures of *M. galloprovincialis* and *P. canalicus* were accessed through the World Register of Marine Species [57,58].

The RAxML-NG phylogenomic reconstruction based on the gene-partitioned concatenated nucleotide alignment of 13 mitochondrial protein-coding genes recovered LGM-781 and LGM-885 within the *P. viridis* clade, together with the complete public mitogenomes of the species listed in Supplementary Table S4 (Figure 7). *Mytilus galloprovincialis* was used as an outgroup because it is a mytilid outside the genus *Perna*, providing an external reference for rooting the tree and interpreting relationships among the *Perna* mitogenomes. The Brazilian assemblies were clearly separated from *P. canaliculus* and *P. perna*, consistent with the BLASTn validation, whole-mitogenome pairwise comparisons, and gene-order conservation reported above. This placement supports the taxonomic identity of LGM-781 and LGM-885 and confirms their suitability as complete Brazilian mitochondrial references for comparative analyses within *P. viridis*.

The topology also supports the use of complete mitogenomes to distinguish among *Perna* species and thus places newly generated invasive-range records within the available *Perna* mitogenome dataset. However, because complete *P. viridis* mitogenomes remain sparsely represented in public databases, the phylogenomic placement of the Brazilian sequences should be interpreted as taxonomic and comparative support rather than as evidence of geographic origin, invasion route, or population connectivity.

## 4. Discussion

### 4.1. Brazilian P. viridis Mitogenomes in the Southwestern Atlantic Context

The present study expands the available mitochondrial reference data for invasive *P. viridis* by providing the first complete mitochondrial genomes from Brazilian specimens. This is an important advance because the recent spread of *P. viridis* along the Brazilian coast has so far been documented mainly through occurrence records, morphology, and partial mitochondrial markers, especially *COX1* [9,13,19,20]. By generating complete, read-supported, and curated mitogenomes from Santa Catarina, the present work extends the available molecular evidence from species confirmation to complete-mitogenome comparison.

The sequenced specimens are part of the broader American invasion context summarized in Figure 1 and Supplementary Table S5, in which Brazilian records form the largest subset. Santa Catarina is relevant because it represents the currently documented southern Brazilian invasion front and includes areas where mollusk farming and coastal biosecurity concerns overlap [20]. The occurrence of *P. viridis* in southern Brazil should be interpreted within a combination of environmental suitability and human-mediated vectors. Temperature tolerance, coastal circulation, vessel traffic, hull fouling, ballast water, port and marina infrastructure, and aquaculture structures are plausible factors for establishment or secondary spread in this sector [3,8,9,10,11,19,20]. However, the present study did not test these ecological mechanisms; they are mentioned only to define the regional context of the two sequenced specimens.

The principal contribution of these mitogenomes is to provide curated Brazilian reference sequences for future comparisons, not to resolve the geographic origin or route of introduction of *P. viridis* in Brazil. Complete mitogenomes allow comparison of full mitochondrial haplotypes, genome architecture, annotation boundaries, and variable regions in a genus for which public complete mitogenomes remain limited [5,23,24,26,27].

### 4.2. Why Complete Mitogenomes Matter Beyond COX1-Based Confirmation

Mitochondrial *COX1* has been central to the molecular confirmation of *P. viridis* in Brazil. It remains an appropriate first-line marker for species-level identification, especially when juvenile, fouling-associated, or morphologically ambiguous specimens are encountered [9,19,20]. However, *COX1* represents only a small fraction of the mitochondrial genome. Thus, it cannot be used to recover information on gene order, coding-region boundaries, codon usage, intergenic regions, structural organization, or the distribution of informative sites across the complete mitochondrial molecule [22,23]. These additional layers of information become increasingly relevant when the objective shifts from confirming occurrence to a complete mitogenome comparison between *P. viridis* and related *Perna* records.

The pairwise comparisons reported here illustrate the added value. Although overall identity was high, complete mitogenome comparisons retained 20 to 125 SNPs/mismatches and one to 12 indel events across approximately 16 kb (Table 3), exceeding the comparative space available from a single *COX1* fragment, while remaining insufficient to infer source populations or invasion routes.

Studies in other invasive or biosecurity-relevant taxa have similarly used complete mitogenomes to recover SNPs, assess structural variation, support marker development, or reconstruct invasion history when broader sampling is available [59,60,61,62,63,64,65]. In *P. viridis*, the complete mitogenomes reported here should therefore be viewed as complementary to *COX1*, broadening the molecular basis for future marker selection and haplotype screening without replacing population-scale sampling.

### 4.3. Read-Supported Assembly and Curated Annotation as Requirements for Reliable Comparative Mitogenomics

The importance of a complete mitogenome for comparative analyses depends not only on obtaining a circular sequence but also on the confidence with which that sequence is assembled and annotated. Long-read ONT-based approaches are increasingly used to recover complete mitochondrial genomes [66,67,68,69]. In the present study, the selected long-amplicon consensuses were supported by 956 to 3918 reads retained in the selected clusters per fragment (Table 2), providing strong sequence support for both Brazilian specimens and reinforcing their value as comparative resources rather than simple species-confirmation sequences.

That level of curation matters because circular mitochondrial assemblies derived from overlapping long-range PCR fragments must reconcile fragment overlap, terminal redundancy, and local sequence conflicts before the final molecule is represented in linearized form [68]. The issue is especially relevant in compact metazoan mitogenomes, where neighboring genes may overlap, and coding boundaries are not always straightforward to define [70,71,72]. In mollusks and mytilid bivalves, these challenges are compounded by lineage-specific structural patterns, rearranged gene orders, and variable intergenic organization [23,24,25]. For that reason, overlap-aware merging, read-supported conflict resolution, and trimming of terminal redundancy must be treated as quality-control steps, not merely as computational formalities.

Annotation required the same level of care. Mytilid mitogenomes may differ in gene order, strand organization, intergenic structure, feature boundaries, and, in some bivalve lineages, mitochondrial inheritance patterns, so automatic annotation or direct transfer from a single reference is often insufficient [23,24,73,74]. The reference-assisted nucleotide-level annotation and manual curation adopted here were therefore necessary to assess coding continuity, start and stop codons, indel effects, and the consistency of protein-coding genes under the invertebrate mitochondrial genetic code. This was especially important for *ND4*, which crosses the artificial coordinate break of the *ATP6*-start representation and was therefore retained as a single origin-spanning CDS, preserving biological continuity rather than truncating the feature at the linearized boundary [26,55].

The final annotations of LGM-781 and LGM-885 were internally coherent, and recovering the same feature set in both specimens, comprising 13 protein-coding genes, 23 tRNAs, two rRNAs, and 38 total mitochondrial features, together with identical A+T content and closely similar intergenic and overlap profiles (Table 4). Agreement between two independently assembled Brazilian specimens is important because comparative work in Mytilidae depends on reliable gene boundaries, strand assignment, and structure-aware annotation, particularly when interspecific rearrangements are present [23,24,27]. These checks support the use of LGM-781 and LGM-885 in comparative analyses.

### 4.4. Structural and Sequence-Level Variation Reveal the Comparative Value of Perna Mitogenomes

The comparison of mitochondrial gene order reinforces the value of complete mitogenomes as structural markers. The two Brazilian assemblies, LGM-781 and LGM-885, shared the same *ATP6*-start organization observed in the two publicly available complete *P. viridis* mitogenomes, whereas *P. canaliculus* and *P. perna* displayed a distinct arrangement involving, among other regions, *ND2*, *ND4*, *ATP8*, *COX1*, and adjacent tRNA blocks (Figure 3; Supplementary Table S4). This pattern is consistent with previous comparative work, demonstrating that mytilid and *Perna* mitogenomes can differ substantially in gene order and that these differences are relevant for both evolutionary interpretation and annotation quality control [23,24,27]. The conservation of organization among the four *P. viridis* mitogenomes considered here, therefore, supports the structural coherence of the Brazilian assemblies within the current species-level dataset.

At the nucleotide level, the sliding-window analysis indicates that variation within the currently available complete *P. viridis* dataset is not evenly distributed along the mitochondrial genome (Figure 5). Diversity remained low across substantial intervals but increased in discrete regions, such as parts of the *ND6*/*ND5* region and the *COX1*-*ND4* sector. Given that only four complete *P. viridis* mitogenomes are available, these regions should be interpreted only as variable intervals that may guide future marker testing after broader sampling.

Protein-level comparisons added a complementary layer, allowing homologous genes to be compared across *Perna* independently of whole-genome collinearity. This distinction matters because the sliding-window analysis is restricted to *P. viridis,* while the rearrangements observed among congeneric species make direct position-to-position mitogenome comparisons less straightforward across the genus. In that gene-wise analysis, *COX1* and *COX2* were among the most conserved proteins, whereas *ATP8* and *ND6* showed the highest mean amino acid divergence, with additional elevated divergence in *ND5*, *ND2*, *ND3*, *ND4L*, and *ND4* (Figure 6). This pattern underscores gene-specific heterogeneity in mitochondrial divergence across *Perna*, but it should not be overinterpreted as evidence of adaptive divergence in the absence of explicit tests.

Phylogenomic reconstruction integrates these structural and sequence-based results. The two Brazilian mitogenomes clustered within the *P. viridis* clade and were clearly separated from *P. canaliculus* and *P. perna* (Figure 7), agreeing with the BLASTn validation, pairwise whole-mitogenome comparisons, and structural conservation summarized above. This placement supports the taxonomic assignment of LGM-781 and LGM-885 and confirms their usefulness for comparative analyses within *P. viridis*. The different closest matches observed for the two Brazilian assemblies should be treated as hypothesis-generating only, not as a resolved biogeographic signal.

### 4.5. Applications, Limitations, and Future Directions

The mitogenomes generated here can support interpretation of future *P. viridis* records from ports, marinas, aquaculture facilities, fouling communities, and biodiversity surveys, particularly when specimens are juvenile, degraded, or morphologically ambiguous. They may also help select mitochondrial targets for future barcoding, metabarcoding, quantitative polymerase chain reaction (qPCR), or droplet digital polymerase chain reaction (ddPCR) assays. Although the two Brazilian assemblies doubled the number of non-redundant complete *P. viridis* mitogenomes available for comparison, the main limitations are equally clear. Only two Brazilian specimens were sequenced, both from Santa Catarina, and the number of complete public *P. viridis* mitogenomes available for comparison remains very small. In addition, mitochondrial data represent a single organellar marker, and mitochondrial inheritance in bivalves, including mytilids, can deviate from strict maternal inheritance through doubly uniparental inheritance in some lineages [73,74]; therefore, these data cannot capture the full demographic complexity of an invasion process. Without broader population sampling, nuclear markers, and denser mitogenome representation from native and invaded regions, the current dataset cannot robustly distinguish between recurrent introduction, secondary dispersal, founder effects, or unsampled source diversity.

Future work should expand sampling across native and invaded regions and integrate complete mitogenomes with nuclear or genome-wide markers, environmental DNA, and information on ports, vessel movement, oceanography, aquaculture, and biofouling. The Brazilian assemblies reported here provide the first complete mitochondrial references for *P. viridis* in Brazil, but they do not substitute for population-scale sampling.

## 5. Conclusions

This study provides the first complete mitochondrial genomes of invasive *P. viridis* from Brazil and the southwestern Atlantic, generating two curated mitochondrial references from Santa Catarina, currently the southernmost documented sector of the Brazilian range. The read-supported assemblies and manually curated annotations extend beyond *COX1*-based confirmation by documenting mitochondrial architecture, gene-order conservation within *P. viridis*, structural divergence among *Perna*, localized nucleotide variability, protein-level heterogeneity, and phylogenomic placement. The Brazilian sequences may support taxonomic validation, marker selection, and future comparative studies across the Brazilian coast and the broader American invasion context. However, the inclusion of only two Brazilian specimens and the scarcity of complete public *P. viridis* mitogenomes mean that these data should be treated as initial comparative references, not as evidence of source populations, invasion routes, or connectivity. Future work should integrate broader native and invaded ranges, nuclear or genome-wide markers, environmental DNA, and information on ports, vessel routes, oceanography, aquaculture, and biofouling to test the processes shaping the expansion of *P. viridis* in the southwestern Atlantic.

## Figures and Tables

**Table 1:** Long-range PCR primers used for two-amplicon amplification of the *Perna viridis* mitochondrial genome. Both specimens were amplified using the same primer pairs. Primer sequences are reported in the 5′→3′ orientation.

Amplicon	Primer Name	Direction	Sequence (5′→3′)
mtDNA Frag-1	Pvir_mtDNA_Frag1_F	Forward	GTGTATCAGTTCAAGTGTGTGG
mtDNA Frag-1	Pvir_mtDNA_Frag1_R	Reverse	AACCTCCTGCAACCATAATCTC
mtDNA Frag-2	Pvir_mtDNA_Frag2_F	Forward	TAGTTGAATCGTGGAGGAAGTC
mtDNA Frag-2	Pvir_mtDNA_Frag2_R	Reverse	CACACACCTACAATTAATGGCC

**Table 2:** Oxford Nanopore reads support and consensus length for each long-range PCR amplicon. Read counts correspond to reads retained in the selected clusters supporting each consensus sequence; consensus length is reported in base pairs (bp).

Sample ID	Amplicon	Reads (n)	Consensus Length (bp)
LGM781	Pvir_mtDNA_Frag-1.1	3918	8463
LGM781	Pvir_mtDNA_Frag-2.1	1354	8658
LGM885	Pvir_mtDNA_Frag-1.2	1773	8459
LGM885	Pvir_mtDNA_Frag-2.2	956	8659

**Table 3:** Compact pairwise comparison among complete mitochondrial genomes included in the analysis. Pairwise identity was estimated from comparable A/C/G/T sites. Indel events correspond to consecutive gap blocks, whereas indel bases correspond to the total number of gap positions within those blocks.

Comparison	Identity (%)	SNPs/ Mismatches	Indel Events	Indel Bases	Comparable Sites
NC_018362.1 vs. MW727515.1	99.875	20	1	1	16,012
LGM-781_v1 vs. MW727515.1	99.8	32	3	6	16,011
LGM-781_v1 vs. NC_018362.1	99.7	48	4	7	16,010
LGM-885_v1 vs. NC_018362.1	99.319	109	12	17	16,003
LGM-885_v1 vs. MW727515.1	99.256	119	11	16	16,004
LGM-781_v1 vs. LGM-885_v1	99.219	125	9	12	16,007

**Table 4:** Genome composition and architectural features of the Brazilian *Perna viridis* mitochondrial genomes analyzed in this study.

Parameter	LGM-781	LGM-885
Genome size (bp)	16,015	16,011
PCGs	13	13
tRNAs	23	23
rRNAs	2	2
Total annotated features	38	38
Strand distribution	38 H; 0 L	38 H; 0 L
A (%)	27.3	27.4
T (%)	40.2	40.1
C (%)	9.9	9.9
G (%)	22.6	22.5
A+T (%)	67.5	67.5
G+C (%)	32.5	32.5
AT-skew	−0.191	−0.189
GC-skew	0.391	0.387
Total PCG length (bp)	11,221	11,218
Total tRNA length (bp)	1522	1523
Total rRNA length (bp)	1935	1934
Intergenic regions	26	26
Total intergenic length (bp)	1356	1355
Largest intergenic region	661 bp (*COX1*–*ND4*)	660 bp (*COX1*–*ND4*)
Overlaps	5	5
Total overlap length (bp)	19	19
Largest overlap	7 bp (*ND4*–*ATP6*; *ND2*–*tRNA-Glu*)	7 bp (*ND4*–*ATP6*; *ND2*–*tRNA-Glu*)
Origin-spanning gene(s)	*ND4*	*ND4*
Gene density (features/kb)	2.373	2.373
Mean bp per feature	421.4	421.3

Note: PCGs = protein-coding genes. Intergenic and overlapping regions were computed using circular coordinates, with origin-spanning features treated as continuous across the circular junction. The largest intergenic region is reported descriptively as a non-coding/intergenic interval and was not assigned as a D-loop, control region, or replication origin. Gene density is shown as features/kb.

## Data Availability

Data will be made available upon reasonable request.

## Supplementary Materials

The following supporting information can be downloaded at: https://www.mdpi.com/article/10.3390/biology15141199/s1, Table S1. PCR primer properties and predicted amplicon information for amplification of the mitochondrial reference sequence of *Perna viridis* (NC_018362.1). Table S2. Read-level support for long-range PCR amplicons used in mitochondrial genome assembly of *Perna viridis*. Table S3. Software, scripts, and key parameters used for reading processing, mitogenome assembly, annotation, comparative analyses, and phylogenetic reconstruction of *Perna viridis* mitochondrial genomes. Table S4. GenBank accession numbers, species, sampling localities (country), and source of locality information for mitogenomes included in the phylogenetic dataset. Sequences retrieved from NCBI GenBank/RefSeq are listed below. Table S5. Literature and data sources used to compile the non-native occurrence records of *Perna viridis* are shown in Figure 1. Source-oriented version. The table summarizes only the references used to support mapped occurrence records, their geographic scope, represented localities, occurrence years, and number of mapped points. Coordinates and internal curation notes were intentionally omitted. Table S6. Summary of the 20 highest-ranked NCBI BLASTN hits for the mitochondrial genome sequence of *Perna viridis* (LGM-781_assembled). Results were obtained using the complete mitochondrial query sequence (16,015 bp) against the NCBI nucleotide collection (nt). The table reports hit description, scores, query coverage, E-value, percentage identity, and accession numbers as presented in the BLAST description table. Hits are shown in the order returned by BLAST. Table S7. Summary of the 20 highest-ranked NCBI BLASTN hits for the mitochondrial genome sequence of *Perna viridis* (LGM-885_assembled). Results were obtained using the complete mitochondrial query sequence (16,011 bp) against the NCBI nucleotide collection (nt). The table reports hit description, scores, query coverage, E-value, percentage identity, and accession numbers as presented in the BLAST description table. Hits are shown in the order returned by BLAST. Table S8. Pairwise BLAST comparison between the mitochondrial genome sequences of *Perna viridis* LGM-781 and LGM-885. The table summarizes the BLAST 2 sequences alignment ranges detected in the direct pairwise analysis, reporting query and subject coordinates, score, E-value, identities, gaps, and strand orientation. Table S9. Mitochondrial gene annotation and coordinates for *Perna viridis* (LGM-781). The table lists all annotated mitochondrial features (13 protein-coding genes, 23 tRNAs, and 2 rRNAs), reporting genomic start and end coordinates, feature length (bp), initiation and termination codons for protein-coding genes, and strand assignment. For non-coding features (tRNAs, rRNAs, and control-region features), start/stop codon fields are left blank because these genes are not translated. Strand orientation is indicated as H (heavy strand) or L (light strand). Table S10. Mitochondrial gene annotation and coordinates for *Perna viridis* (LGM-885). The table lists all annotated mitochondrial features (13 protein-coding genes, 23 tRNAs, and 2 rRNAs), reporting genomic start and end coordinates, feature length (bp), initiation and termination codons for protein-coding genes, and strand assignment. For non-coding features (tRNAs, rRNAs, and control-region features), start/stop codon fields are left blank because these genes are not translated. Strand orientation is indicated as H (heavy strand) or L (light strand).
