# Supplementary material for: First Complete Mitochondrial Genomes of the Invasive Mussel Perna viridis from Brazil and the Southwestern Atlantic
Source: Biology (Basel). 2026 Jul 20;15(14):1199. doi: 10.3390/biology15141199 (PMC13404536; doi:10.3390/biology15141199)
Supplement: Supplementary file 1 [file biology-15-01199-s001.zip › biology-4397583-supplementary.pdf]

Table S1. PCR primer properties and predicted amplicon information for amplification of the mitochondrial reference sequence of *Perna viridis* (NC_018362.1).

A. Primer-level properties

Amplicon	Primer name	Direction	Sequence (5′→3′)	Length (nt)	GC (%)	Tm (°C)	Self-dimer ΔG (kcal/mol)	Hairpin ΔG (kcal/mol)	Reference coordinates (1-based)
mtDNA Frag-1	Pvir_mtDNA_Frag1_F	Forward	GTGTATCAGTTCAAGTGTGTGG	22	45.5	55.13	-2.036	not detected	11057-11078
mtDNA Frag-1	Pvir_mtDNA_Frag1_R	Reverse	AACCTCCTGCAACCATAATCTC	22	45.5	55.97	-2.898	not detected	3526-3547
mtDNA Frag-2	Pvir_mtDNA_Frag2_F	Forward	TAGTTGAATCGTGGAGGAAGTC	22	45.5	55.41	-1.902	not detected	2785-2806
mtDNA Frag-2	Pvir_mtDNA_Frag2_R	Reverse	CACACACCTACAATTAATGGCC	22	45.5	55.49	-4.541	not detected	11469-11490

B. Amplicon-level summary

Amplicon	Forward primer	Reverse primer	Expected amplicon length (bp)	Amplicon coordinates in reference (1-based)	Crosses circular origin	Pair heterodimer ΔG (kcal/mol)
mtDNA Frag-1	Pvir_mtDNA_Frag1_F	Pvir_mtDNA_Frag1_R	8505	11057→3547	Yes	-2.428
mtDNA Frag-2	Pvir_mtDNA_Frag2_F	Pvir_mtDNA_Frag2_R	8706	2785-11490	No	-4.153

* Tm, GC content, ΔG metrics, primer coordinates, and theoretical amplicon lengths were calculated from primer sequences and the reference mitogenome NC_018362.1. 'not detected' indicates that the hairpin calculation was performed. Still, no hairpin was detected under the evaluated conditions. Coordinates are 1-based and include primer binding sites; start → end indicates that the predicted amplicon crosses the circular origin.

Table S2. Read-level support for long-range PCR amplicons used in mitochondrial genome assembly of *Perna viridis*.

Sample ID	Amplicon	Reads after in silico PCR (n)	Reads in selected clusters (n)	Retained reads (%)	Mean read length (bp)	Median read length (bp)	Read length N50 (bp)	Read length range (bp)
LGM-781	mtDNA Frag-1	4,322	3,918	90.7	8,441.94	8,446	8,446	8,209-8,970
LGM-781	mtDNA Frag-2	1,623	1,354	83.4	8,641.52	8,644	8,644	8,516-8,818
LGM-885	mtDNA Frag-1	2,429	1,773	73.0	8,428.45	8,433	8,433	8,291-8,989
LGM-885	mtDNA Frag-2	1,175	956	81.4	8,634.60	8,638	8,638	8,470-8,754
Total	-	9,549	8,001	83.8	-	-	-	-

* Reads after *in silico* PCR correspond to quality-filtered and demultiplexed reads recovered by virtual PCR for each amplicon. Selected clusters refer to the two highest-supported read clusters retained for consensus generation. Retained reads (%) = selected-cluster reads/reads after *in silico* PCR x 100. N50 was calculated from read lengths in the selected clusters.

Table S3. Software, scripts, and key parameters used for reading processing, mitogenome assembly, annotation, comparative analyses, and phylogenetic reconstruction of *Perna viridis* mitochondrial genomes.

Workflow step	Tool / script	Key parameters and settings	Output or use in this study
Basecalling and read filtering	MinKNOW v25.03.9; Dorado (Oxford Nanopore Technologies)	Basecalling in super-accurate mode; minimum read quality threshold Q >= 8; FASTQ output retained for downstream processing.	Quality-filtered Nanopore reads used for demultiplexing, in silico PCR recovery, and consensus generation.
Barcode demultiplexing and amplicon read recovery	Custom InSilicoPCR_GUI_V4G_v7 workflow; BBMap msa.sh; cutprimers.sh; SeqKit	Sample-identifying 5-nt barcodes matched at 100% identity. Primer matching identity threshold = 85%. BBMap msa.sh search used qin=33, reverse-complement search enabled (rcomp=t), address reporting enabled (addr=t), and replicate hits allowed (replicate=t). Primer-trimmed reads were extracted with cutprimers.sh and length-filtered with SeqKit using amplicon-specific size limits.	Demultiplexed and amplicon-recovered reads were used as input for clustering and consensus generation; read-level summaries are reported in Table S2.
Read clustering	CD-HIT v4.8.1, called within ConsensusCalculator workflow	cd-hit parameters: -c 0.7, -n 3, -aL 0.8, -aS 0.8, -d 100, -T 10. Consensus generation required a minimum of 5 reads per cluster.	Read clusters used to identify the highest-supported long-amplicon consensus for each specimen and fragment.
Cluster alignment	MAFFT v7.520, called within ConsensusCalculator workflow	mafft --quiet --thread 10; no additional manual fine-tuning was applied.	Multiple-sequence alignments of reads within selected clusters for consensus construction.
Consensus generation	EMBOSS v6.6.0.0; cons program	EMBOSS Cons was applied to MAFFT alignments using default consensus-generation settings in the workflow.	Consensus sequences for each selected long-range PCR amplicon.
Overlap-based mitogenome assembly and conflict resolution	Custom Python assembly/curation workflow	Terminal overlaps were identified using minimum overlap = 300 bp, identity >= 95%, and comparable bases >= 50%. Fragments were merged by majority rule; ambiguous positions were assigned as N when base support was insufficient. Conflict resolution used original long reads with reverse-complement matching, 15-bp flanks, 13-bp seed size, maximum mismatch threshold = 18%, and up to 500 supporting reads per position. Terminal redundancy was trimmed using the same overlap criteria.	Complete circular mitochondrial genome assemblies for LGM-781 and LGM-885.
Taxonomic validation by similarity search	NCBI BLASTn; BLAST 2 sequences where applicable	Complete mitogenome sequences were queried against the NCBI nucleotide/Core nucleotide database. Top BLASTn hits and direct pairwise BLAST outputs were retained for supplementary tables.	Taxonomic validation of the final assemblies and supplementary BLAST summaries (Tables S6A-S6C).
Pairwise mitogenome comparison	Custom Python pairwise-comparison script	Pairwise identity calculated only from comparable A/C/G/T sites. SNPs/mismatches were counted across comparable sites. Consecutive gap blocks were counted as indel events, and total gap positions within those blocks were counted as indel bases.	Compact pairwise comparison among complete *P. viridis* mitogenomes (Table 3).
Reference-assisted annotation and manual curation	Custom mitog_v130_corrigido workflow; *P. viridis* RefSeq NC_018362.1; manual inspection	Reference-assisted nucleotide-level comparison with minimum alignment identity = 85%. PairwiseAligner settings included mismatch score = -1, open gap score = -2, and extend gap score = -0.5. Coding sequences were checked with the invertebrate mitochondrial genetic code (translation table 5). Gene boundaries, reading frames, start/stop codons, circular continuity, and intergenic/overlap intervals were manually inspected.	Curated gene annotations, coordinates, feature lengths, strand assignments, and coding-region checks (Tables S7A-S7B).
Circular genome visualization	MitoDraw module of MitoFish	Curated GBK files were used as input for circular representation. No additional analytical parameter was changed beyond using the manually curated annotation as input.	Representative circular mitochondrial map (Figure 2).
Gene-order schematic comparison	Custom Python parsing of GenBank/GBK annotations	CDS, tRNA, and rRNA features were parsed from GBK annotations and represented as comparative blocks using an *ATP6*-start orientation for direct visualization.	Gene-order comparison among *P. viridis*, *P. perna*, and *P. canaliculus* mitogenomes (Figure 3).
Relative synonymous codon usage (RSCU)	PhyloSuite v1.2.3	RSCU was calculated from extracted mitochondrial protein-coding genes using the standard/default RSCU workflow in PhyloSuite; no custom parameter modification was applied.	Codon-usage profile of the representative Brazilian mitogenome LGM-781 (Figure 4).
Sliding-window nucleotide diversity	Clustal Omega; custom Python sliding-window script	Complete *P. viridis* mitogenomes were aligned in DNA mode using Clustal Omega --auto. Nucleotide diversity was calculated with a window = 500 bp and step = 25 bp. Alignment columns with >50% gaps were excluded, and windows retained positions with valid DNA calls (A/C/G/T) in at least 50% of sequences.	Genome-wide sliding-window nucleotide-diversity profile across complete *P. viridis* mitogenomes (Figure 5).
Protein-level divergence	Custom extraction/comparison workflow	Amino acid sequences were extracted for each mitochondrial protein-coding gene, aligned by gene, and compared across *Perna* mitogenomes at comparable amino acid positions.	Mean amino acid divergence by mitochondrial protein-coding gene (Figure 6).
Phylogenomic reconstruction	RAxML-NG v2.0.2; iTOL for visualization	The 13 mitochondrial protein-coding genes were aligned separately and concatenated in the order *COX1*, *COX2*, *COX3*, *ATP6*, *ATP8*, *CYTB*, *ND1*, *ND2*, *ND3*, *ND4*, *ND4L*, *ND5*, and *ND6*. Final nucleotide alignment: 15 sequences and 12,051 positions. Dataset partitioned by gene (13 partitions). Models GTR+FC+G4m are estimated for each partition. Analysis used three starting trees and 1,000 Felsenstein bootstrap replicates. The best-scoring ML tree was visualized in iTOL and rooted using *Mytilus galloprovincialis* OR574991.1 as an outgroup.	RAxML-NG phylogenomic tree used as the revised Figure 7.

Note: For custom scripts and workflows, the table lists the parameters that directly affect read selection, consensus generation, assembly, annotation, and comparative analyses. Default settings are indicated only when no custom parameter was changed by the user. PCG = protein-coding gene; GBK = GenBank flat file; ML = maximum likelihood.

Table S4. GenBank accession numbers, species, sampling localities (country), and source of locality information for mitogenomes included in the phylogenetic dataset. Sequences retrieved from NCBI GenBank/RefSeq are listed below.

Species	NCBI - GenBank	Country	Source [Reference number]*
Mytilus galloprovincialis	OR574991.1	Australia	[38]
Perna canaliculus	MG766134.1	New Zealand	[39]
Perna canaliculus	MK775557.1	New Zealand	[40]
Perna canaliculus	MK775558.1	New Zealand	[40]
Perna canaliculus	MW727514.1	Australia	[41]
Perna canaliculus	NC_054242.1	New Zealand	[40]
Perna perna	MT588202.1	N/A	[42]
Perna perna	OK576479.1	Brazil	[24]
Perna perna	OK576480.1	South Africa	[24]
Perna perna	OK576481.1	South Africa	[24]
Perna perna	PP059121.1	South Africa	[43]
Perna viridis	MW727515.1	Australia	[56]
Perna viridis	NC_018362.1	China	[26]

* GenBank = sampling country retrieved directly from GenBank/RefSeq source information. Literature citations indicate the locality retrieved from the cited publication. N/A = not available.

Table S5. Literature and data sources used to compile the non-native occurrence records of *Perna viridis* are shown in Figure 1. Source-oriented version. The table summarizes only the references used to support mapped occurrence records, their geographic scope, represented localities, occurrence years, and number of mapped points. Coordinates and internal curation notes were intentionally omitted.

[redacted]				
[48,50]	Trinidad and Tobago	Point Lisas / Gulf of Paria, Trinidad	1990	1
[51,53]	Venezuela	Gulf of Paria / northeastern Venezuela	1993	1
[48,52]	Jamaica	Kingston Harbour, Jamaica	1998	1
[53,56]	United States	Southeastern USA records, including Florida, Georgia, South Carolina, and the Alabama/Florida sector	1999, 2001, 2002, 2003, 2005, 2006, 2008, 2011	12
[12,49]	Colombia	Cartagena Bay port area and Virgen Marsh / Bocana sector, Cartagena	2009, 2020	2
[55]	Colombia	Cispatá Bay, Puerto Velero, and Vía Parque Isla de Salamanca, Colombian Caribbean	2013, 2018	3
[13]	Brazil — Rio de Janeiro	Guanabara Bay, Rio de Janeiro	2018	1
[14]	Brazil — Rio de Janeiro	Arraial do Cabo Marine Extractive Reserve, Rio de Janeiro	2022	1
[19]	Brazil — Paraná	Ponta do Poço Marina Club, Paranaguá Estuarine Complex, Paraná	2023	1
[18]	Brazil — Rio de Janeiro, São Paulo, Paraná, and Santa Catarina	Multiple Brazilian coastal records, including port, mariculture, rocky-shore, beach, and monitoring localities	2022–2024	49
[17]	Brazil — São Paulo	São Paulo coast, including Santos Bay, Bom Abrigo Island, Cananeia, São Sebastião, Caraguatatuba, Ilha do Cardoso, Iguape, and adjacent localities	2023	10
[16]	Brazil — São Paulo	Saco da Ribeira Pier, Ubatuba, São Paulo	2024	1
[20]	Brazil — Santa Catarina	Itapocoroy Bay / Penha, Santa Catarina	2024	1

Table S6. Summary of the 20 highest-ranked NCBI BLASTN hits for the mitochondrial genome sequence of *Perna viridis* (LGM-781_assembled). Results were obtained using the complete mitochondrial query sequence (16015 bp) against the NCBI nucleotide collection (nt). The table reports hit description, scores, query coverage, E-value, percentage identity, and accession numbers as presented in the BLAST description table. Hits are shown in the order returned by BLAST.

Rank	BLAST hit description	Max score	Total score	Query cover	E-value	Identity	Accession
1	*Perna viridis* mitochondrion, complete genome	29362	29486	100%	0.0	99.76%	MW727515.1
2	*Perna viridis* mitochondrion, complete genome	29264	29389	100%	0.0	99.65%	NC_018362.1
3	*Perna viridis* isolate Ven17 cytochrome oxidase subunit I (cox1) gene, partial cds; mitochondrial	2383	2383	8%	0.0	99.69%	DQ343589.1
4	*Perna viridis* isolate US16 cytochrome oxidase subunit I (cox1) gene, partial cds; mitochondrial	2370	2370	8%	0.0	99.69%	DQ343587.1
5	*Perna viridis* haplotype Hap11 cytochrome c oxidase subunit I gene, partial cds; and D-loop, complete sequence; mitochondrial	2233	2233	8%	0.0	99.75%	MH285349.1
6	*Perna viridis* haplotype Hap34 cytochrome c oxidase subunit I gene, partial cds; and D-loop, complete sequence; mitochondrial	2217	2217	8%	0.0	99.51%	MH285372.1
7	*Perna viridis* haplotype Hap26 cytochrome c oxidase subunit I gene, partial cds; and D-loop, complete sequence; mitochondrial	2217	2217	8%	0.0	99.51%	MH285364.1
8	*Perna viridis* haplotype Hap12 cytochrome c oxidase subunit I gene, partial cds; and D-loop, complete sequence; mitochondrial	2217	2217	8%	0.0	99.51%	MH285350.1
9	*Perna viridis* haplotype Hap9 cytochrome c oxidase subunit I gene, partial cds; and D-loop, complete sequence; mitochondrial	2217	2217	8%	0.0	99.51%	MH285347.1
10	*Perna viridis* haplotype Hap8 cytochrome c oxidase subunit I gene, partial cds; and D-loop, complete sequence; mitochondrial	2217	2217	8%	0.0	99.51%	MH285346.1
11	*Perna viridis* haplotype Hap37 cytochrome c oxidase subunit I gene, partial cds; and D-loop, complete sequence; mitochondrial	2211	2211	8%	0.0	99.43%	MH285375.1
12	*Perna viridis* haplotype Hap36 cytochrome c oxidase subunit I gene, partial cds; and D-loop, complete sequence; mitochondrial	2211	2211	8%	0.0	99.43%	MH285374.1
13	*Perna viridis* haplotype Hap35 cytochrome c oxidase subunit I gene, partial cds; and D-loop, complete sequence; mitochondrial	2211	2211	8%	0.0	99.43%	MH285373.1
14	*Perna viridis* haplotype Hap27 cytochrome c oxidase subunit I gene, partial cds; and D-loop, complete sequence; mitochondrial	2211	2211	8%	0.0	99.43%	MH285365.1
15	*Perna viridis* haplotype Hap23 cytochrome c oxidase subunit I gene, partial cds; and D-loop, complete sequence; mitochondrial	2211	2211	8%	0.0	99.43%	MH285361.1
16	*Perna viridis* haplotype Hap15 cytochrome c oxidase subunit I gene, partial cds; and D-loop, complete sequence; mitochondrial	2211	2211	8%	0.0	99.43%	MH285353.1
17	*Perna viridis* haplotype Hap13 cytochrome c oxidase subunit I gene, partial cds; and D-loop, complete sequence; mitochondrial	2211	2211	8%	0.0	99.43%	MH285351.1
18	*Perna viridis* haplotype Hap10 cytochrome c oxidase subunit I gene, partial cds; and D-loop, complete sequence; mitochondrial	2211	2211	8%	0.0	99.43%	MH285348.1
19	*Perna viridis* haplotype Hap4 cytochrome c oxidase subunit I gene, partial cds; and D-loop, complete sequence; mitochondrial	2211	2211	8%	0.0	99.43%	MH285342.1
20	*Perna viridis* haplotype Hap2 cytochrome c oxidase subunit I gene, partial cds; and D-loop, complete sequence; mitochondrial	2211	2211	8%	0.0	99.43%	MH285340.1

***** All listed hits were reported by BLAST as *Perna viridis*. Query cover and percent identity are reported exactly as shown in the NCBI BLAST output. Only the first 20 hits from the NCBI BLAST description table are included.

Table S7. Summary of the 20 highest-ranked NCBI BLASTN hits for the mitochondrial genome sequence of *Perna viridis* (LGM-885_assembled). Results were obtained using the complete mitochondrial query sequence (16011 bp) against the NCBI nucleotide collection (nt). The table reports hit description, scores, query coverage, E-value, percentage identity, and accession numbers as presented in the BLAST description table. Hits are shown in the order returned by BLAST.

Rank	BLAST hit description	Max score	Total score	Query cover	E-value	Identity	Accession
1	*Perna viridis* mitochondrion, complete genome	28906	29030	100%	0.0	99.25%	NC_018362.1
2	*Perna viridis* mitochondrion, complete genome	28860	28984	100%	0.0	99.20%	MW727515.1
3	*Perna viridis* isolate Ven17 cytochrome oxidase subunit I (cox1) gene, partial cds; mitochondrial	2333	2333	8%	0.0	99.00%	DQ343589.1
4	*Perna viridis* isolate US16 cytochrome oxidase subunit I (cox1) gene, partial cds; mitochondrial	2320	2320	8%	0.0	99.00%	DQ343587.1
5	*Perna viridis* haplotype Hap6 cytochrome c oxidase subunit I gene, partial cds; and D-loop, complete sequence; mitochondrial	2220	2220	8%	0.0	99.59%	MH285344.1
6	*Perna viridis* haplotype Hap7 cytochrome c oxidase subunit I gene, partial cds; and D-loop, complete sequence; mitochondrial	2215	2215	8%	0.0	99.51%	MH285345.1
7	*Perna viridis* haplotype Hap5 cytochrome c oxidase subunit I gene, partial cds; and D-loop, complete sequence; mitochondrial	2209	2209	8%	0.0	99.43%	MH285343.1
8	*Perna viridis* haplotype Hap24 cytochrome c oxidase subunit I gene, partial cds; and D-loop, complete sequence; mitochondrial	2204	2204	8%	0.0	99.34%	MH285362.1
9	*Perna viridis* haplotype Hap18 cytochrome c oxidase subunit I gene, partial cds; and D-loop, complete sequence; mitochondrial	2204	2204	8%	0.0	99.34%	MH285356.1
10	*Perna viridis* haplotype Hap16 cytochrome c oxidase subunit I gene, partial cds; and D-loop, complete sequence; mitochondrial	2193	2193	8%	0.0	99.18%	MH285354.1
11	*Perna viridis* haplotype Hap3 cytochrome c oxidase subunit I gene, partial cds; and D-loop, complete sequence; mitochondrial	2193	2193	8%	0.0	99.18%	MH285341.1
12	*Perna viridis* haplotype Hap40 cytochrome c oxidase subunit I gene, partial cds; and D-loop, complete sequence; mitochondrial	2187	2187	8%	0.0	99.10%	MH285378.1
13	*Perna viridis* haplotype Hap38 cytochrome c oxidase subunit I gene, partial cds; and D-loop, complete sequence; mitochondrial	2187	2187	8%	0.0	99.10%	MH285376.1
14	*Perna viridis* haplotype Hap28 cytochrome c oxidase subunit I gene, partial cds; and D-loop, complete sequence; mitochondrial	2187	2187	8%	0.0	99.10%	MH285366.1
15	*Perna viridis* haplotype Hap21 cytochrome c oxidase subunit I gene, partial cds; and D-loop, complete sequence; mitochondrial	2187	2187	8%	0.0	99.10%	MH285359.1
16	*Perna viridis* haplotype Hap20 cytochrome c oxidase subunit I gene, partial cds; and D-loop, complete sequence; mitochondrial	2187	2187	8%	0.0	99.10%	MH285358.1
17	*Perna viridis* haplotype Hap19 cytochrome c oxidase subunit I gene, partial cds; and D-loop, complete sequence; mitochondrial	2187	2187	8%	0.0	99.10%	MH285357.1
18	*Perna viridis* haplotype Hap14 cytochrome c oxidase subunit I gene, partial cds; and D-loop, complete sequence; mitochondrial	2187	2187	8%	0.0	99.10%	MH285352.1
19	*Perna viridis* haplotype Hap39 cytochrome c oxidase subunit I gene, partial cds; and D-loop, complete sequence; mitochondrial	2182	2182	8%	0.0	99.02%	MH285377.1
20	*Perna viridis* haplotype Hap30 cytochrome c oxidase subunit I gene, partial cds; and D-loop, complete sequence; mitochondrial	2182	2182	8%	0.0	99.02%	MH285368.1

* All listed hits were reported by BLAST as *Perna viridis*. Query cover and percent identity are reported exactly as shown in the NCBI BLAST output. Only the first 20 hits from the NCBI BLAST description table are included.

Table S8. Pairwise BLAST comparison between the mitochondrial genome sequences of *Perna viridis* LGM-781 and LGM-885. The table summarizes the BLAST 2 sequences alignment ranges detected in the direct pairwise analysis, reporting query and subject coordinates, score, E-value, identities, gaps, and strand orientation.

LGM-781 vs LGM-885 - *Perna viridis*						
**Range**	**Coordinates**		**Alignment statistics**			**Orient.**
**Range**	**Query**	**Subject**	**Score**	**E-value**	**Identities**	**Gaps / strand**
**Range 1**	1-16,015	1-16,011	28,851 bits (15,623)	0.0	15,890/16,020 (99%)	14/16,020 (0%) Plus/Plus

* Pairwise comparison obtained with NCBI BLAST 2 sequences. Query: LGM-781 (16,015 bp); subject: LGM-885 (16,011 bp).

Table S9. Mitochondrial gene annotation and coordinates for *Perna viridis* (LGM-781). The table lists all annotated mitochondrial features (13 protein-coding genes, 23 tRNAs, and 2 rRNAs), reporting genomic start and end coordinates, feature length (bp), initiation and termination codons for protein-coding genes, and strand assignment. For non-coding features (tRNAs, rRNAs and control-region features), start/stop codon fields are left blank because these genes are not translated. Strand orientation is indicated as H (heavy strand) or L (light strand).

**LGM-781 - *Perna viridis***						
**Gene**	**Position**		**Length (bp)**	**Codon**		**Strand (H/L)**
	**From**	**To**		**Start**	**Stop**	
**ATPase6**	1	714	714	ATG	TAG	H
tRNA-Ser	720	785	66			H
**Cytb**	787	1938	1152	ATG	TAG	H
tRNA-Ala	1997	2063	67			H
**COXII**	2089	2778	690	GTG	TAG	H
tRNA-Arg	2802	2868	67			H
tRNA-Met	2881	2945	65			H
tRNA-Asp	2964	3028	65			H
**ND1**	3029	3964	936	ATG	TAG	H
**ND4L**	3974	4282	309	ATT	TAA	H
tRNA-Tyr	4247	4312	66			H
**ND3**	4312	4665	354	ATG	TAG	H
tRNA-Thr	4688	4755	68			H
**ND2**	4818	5828	1011	ATA	TAA	H
tRNA-Glu	5822	5887	66			H
tRNA-Gln	5889	5957	69			H
tRNA-Ser	5955	6021	67			H
tRNA-Pro	6044	6108	65			H
tRNA-Leu	6138	6203	66			H
tRNA-Asn	6221	6283	63			H
**ND6**	6327	6803	477	ATG	TAG	H
tRNA-Cys	6810	6880	71			H
tRNA-Trp	6922	6986	65			H
tRNA-Lys	7036	7103	68			H
**ND5**	7104	8831	1728	ATG	TAG	H
tRNA-Leu	8972	9037	66			H
12S rRNA	9038	9825	788			H
tRNA-Ile	9826	9894	69			H
tRNA-Gly	9897	9962	66			H
tRNA-Val	10002	10068	67			H
16S rRNA	10069	11215	1147			H
tRNA-Met	11216	11279	64			H
tRNA-Phe	11288	11350	63			H
tRNA-His	11356	11418	63			H
**COXIII**	11419	12243	825	ATG	TAG	H
**ATPase8**	12261	12407	147	ATT	TAA	H
**COXI**	12449	13984	1536	ATG	TAA	H
**ND4**	14646; 1	16015; 7	1377	ATG	TAA	H

* Origin-spanning feature: split circular intervals are shown as paired From and To coordinates separated by semicolons, corresponding to the segments before and after the origin of the circular mitochondrial genome.

Table S10. Mitochondrial gene annotation and coordinates for *Perna viridis* (LGM-885). The table lists all annotated mitochondrial features (13 protein-coding genes, 23 tRNAs, and 2 rRNAs), reporting genomic start and end coordinates, feature length (bp), initiation and termination codons for protein-coding genes, and strand assignment. For non-coding features (tRNAs, rRNAs and control-region features), start/stop codon fields are left blank because these genes are not translated. Strand orientation is indicated as H (heavy strand) or L (light strand).

**LGM-885 - *Perna viridis***						
**Gene**	**Position**		**Length (bp)**	**Codon**		**Strand (H/L)**
	**From**	**To**		**Start**	**Stop**	
**ATPase6**	1	714	714	ATG	TAG	H
tRNA-Ser	720	785	66			H
**Cytb**	787	1938	1152	ATG	TAG	H
tRNA-Ala	1997	2063	67			H
**COXII**	2088	2777	690	GTG	TAG	H
tRNA-Arg	2801	2867	67			H
tRNA-Met	2880	2944	65			H
tRNA-Asp	2963	3027	65			H
**ND1**	3028	3963	936	ATG	TAG	H
**ND4L**	3973	4281	309	ATT	TAA	H
tRNA-Tyr	4246	4311	66			H
**ND3**	4311	4664	354	ATG	TAG	H
tRNA-Thr	4687	4754	68			H
**ND2**	4817	5827	1011	ATA	TAA	H
tRNA-Glu	5821	5886	66			H
tRNA-Gln	5890	5958	69			H
tRNA-Ser	5956	6022	67			H
tRNA-Pro	6045	6109	65			H
tRNA-Leu	6139	6204	66			H
tRNA-Asn	6222	6285	64			H
**ND6**	6329	6805	477	ATG	TAG	H
tRNA-Cys	6812	6882	71			H
tRNA-Trp	6924	6988	65			H
tRNA-Lys	7038	7105	68			H
**ND5**	7106	8830	1725	ATG	TAG	H
tRNA-Leu	8971	9036	66			H
12S rRNA	9037	9823	787			H
tRNA-Ile	9824	9892	69			H
tRNA-Gly	9895	9960	66			H
tRNA-Val	10000	10066	67			H
16S rRNA	10067	11213	1147			H
tRNA-Met	11214	11277	64			H
tRNA-Phe	11286	11348	63			H
tRNA-His	11354	11416	63			H
**COXIII**	11417	12241	825	ATG	TAG	H
**ATPase8**	12258	12404	147	ATT	TAA	H
**COXI**	12446	13981	1536	ATG	TAA	H
**ND4**	14642; 1	16011; 7	1377	ATG	TAA	H

* Origin-spanning feature: split circular intervals are shown as paired From and To coordinates separated by semicolons, corresponding to the segments before and after the origin of the circular mitochondrial genome.
